# Detection and Localization of Rabbit Hepatitis E Virus and Antigen in Systemic Tissues from Experimentally Intraperitoneally Infected Rabbits

**DOI:** 10.1371/journal.pone.0088607

**Published:** 2014-03-04

**Authors:** Jingjing Mao, Yue Zhao, Ruiping She, Binbin Cao, Peng Xiao, Qiaoxing Wu, Zhaojie Guo, Longhuan Ma, Majid Hussain Soomro

**Affiliations:** Department of Veterinary Pathology, Laboratory of Veterinary Pathology and Public Health, College of Veterinary Medicine, China Agricultural University, Key Laboratory of Zoonosis of Ministry of Agriculture, Beijing, China; University of North Carolina School of Medicine, United States of America

## Abstract

Rabbit hepatitis E virus (HEV) is a novel genotype of HEV, and is considered to pose a risk of zoonotic transmission. Research into the systemic distribution of rabbit HEV in rabbits during different periods of infection has rarely been reported. To better understand this virus, we infected rabbits with second-passage rabbit HEV via an intraperitoneal route. After inoculation, the infection showed two types, temporary and constant infection. The detection of HEV RNA in the feces varied with time, and serum antigen correlated with fecal HEV RNA. Viremia only appeared 72 days after inoculation. The rabbits remained antibody negative throughout the experimental period. When HEV was localized, several organs besides the liver were HEV RNA positive. Tissue antigen was observed immunohistochemically in the different cells of various organs, especially in parts of the small intestine and the characteristic rabbit gut-associated lymphoid tissue. These data provide valuable information for future research into the pathogenesis of HEV.

## Introduction

Hepatitis E (HE) is an acute self-limiting disease caused by a nonenveloped, positive-sense, single-stranded RNA virus designated “hepatitis E virus” (HEV) [1], [2]. The disease is prevalent in undeveloped countries and in industrialized countries, including Asia and Africa, whereas in developed countries, HE is endemic and sporadic [3]. HEV has three open reading frames (ORFs): ORF1 encodes the nonstructural proteins, ORF2 encodes the capsid protein, and ORF3 encodes a small functional protein that is believed to participate in viral infection and egress and in the regulation of the host responses [1], [4]–[6].

A recent study demonstrated that HE is a zoonotic disease. As well as human HEV, some animal strains of HEV has been discovered, including swine HEV from pigs in 1997 and avian HEV from chickens in 2001 [7], [8]. Several studies have also shown that HEV can be isolated from rats and boars [9], [10]. Recently, rabbit HEV was found in China, with an overall nucleotide similarity of 77–79% to genotypes 1–4 of HEV [11]. The sera of 15.4% of rabbits were positive for HEV antibodies in 9 of 10 counties of China [12]. The United States and France have subsequently reported the presence of rabbit HEV. Serum, liver, bile, intestine, and cecum samples from wild rabbits tested positive for HEV RNA [13], [14]. In view of the prevalence of rabbit HEV and the risk of its zoonotic transmission from rabbits, rabbit HEV warrants more concern as a public health risk [15].

Although rabbit HEV is not highly similar to other mammalian HEV genotypes, a recent experimental study suggested that rabbits can be successfully infected with HEV genotypes 1–4 as well as rabbit HEV [16], indicating that rabbits might be a suitable and readily available animal model for HE. However, the systemic distribution of HEV RNA and antigens is not yet well documented. Moreover, the route of experimental HEV infection in the past has been primarily by intravenous injection, which successfully infects certain animals, such as swine, BALB/c nude mice, and cynomolgus macaques [17]–[19]. However, the use of an intraperitoneal route for the infection of rabbits with HEV is rarely reported and its efficacy is still unclear. The objectives of this study were to investigate whether rabbits can be infected with HEV via an intraperitoneal route and to better understand the distribution and localization of HEV and an HEV antigen.

## Materials and Methods

### Ethics statement

The animal experiments were approved by the Animal Care and Use Committee of China Agricultural University (CAU) (permit number: 20121110-178). We followed the guidelines of the CAU Animal Care and Use Committee in handling the experimental animals during this study.

### Virus

The strain of rabbit HEV was derived from the second passage of a fecal sample from a rabbit infected with rabbit HEV (rhBJ1, accession number KF648530). A 10% suspension of positive feces was prepared [17] and titered with real-time PCR, as described previously [14], [20]. The titer of the suspension was 2.746×10^7^ genome equivalents per mL. The viral suspension was stored at −86°C for later use.

### Animals

Sixteen 80-day-old rabbits were purchased from Xing Long Experimental Animal Center, Beijing, China. Before inoculation, the sera of the rabbits were confirmed to be negative for HEV antibodies with reverse transcription-nested PCR (RT-nPCR) [8], [21]. with outer primers 5′-AATTATGCYCAGTAYCGRGTTG-3′ and 5′-CCCTTRTCYTGCTGMGCATTCTC-3′, and inner primers 5′-GTWATGCTYTGCATWCATGGCT-3′ and 5′-AGCCGACGAAATCAATTCTGTC-3′.

### Experimental design

Sixteen rabbits were randomly divided into two groups, with eight rabbits in each group. Each rabbit in the inoculated group was inoculated intraperitoneally with 5 mL of viral suspension, whereas each rabbit in the control group was injected with an equal volume of phosphate-buffered saline (PBS).

### Sampling

Serum and feces were collected at 1, 4, 7, 14, 21, 28, 35, 42, 52, 62, and 72 days postinoculation (dpi). The serum samples were collected from the marginal ear veins and tested for anti-HEV IgM and IgG antibodies with an enzyme-linked immunosorbent assay (ELISA) based on the HEV ORF2 protein [22], [23]. The serum and feces were tested by RT-nPCR for HEV RNA. The rabbits were necropsied at 14 and 72 dpi. Their livers, lungs, kidneys, spleens, lymph nodes, duodenums, jejunums, ileums, sacculi rotundus (SR), and appendices were collected and stored at −80°C for RT-nPCR analysis.

### ELISAs for HEV antibodies and antigen

All the serum samples were collected and tested for HEV antigen and antibodies, including IgM and IgG antibodies, with an HEV ELISA kits. All assays were performed according to the manufacturer's instructions (Wantai, Beijing, China).

### Immunohistochemical staining for HEV antigen in rabbit tissues

Livers, lungs, kidneys, spleens, lymph nodes, duodenums, jejunums, ileums, SR, and appendices were collected and fixed in 4% paraformaldehyde for 48 h. The fixed tissues were then processed routinely in paraffin and 4-mm sections were prepared. Monoclonal mouse anti-HEV ORF2 antibody (1∶300 dilution; Beijing Protein Institute, Beijing, China) was used as the primary antibody. The primary antibody was added to the sections and incubated at 37°C for 2 h. Immunohistochemical staining was performed according to the instructions of the Histostain™-Plus Kit (ZSGB-BIO, Beijing, China). 3,3′- Diaminobenzidine tetrahydrochloride (DAB; ZSGB-BIO, Beijing, China) was applied for 10 min to visualize the antigen–antibody reaction, and then Gill's hematoxylin was applied as the background stain. The primary antibody was replaced with phosphate-buffered saline in the negative control. The slides were then observed under an Olympus microscope (Japan).

## Results

### Clinical observations

No clinical symptoms were observed in the HEV-infected rabbits. However, one rabbit died during the study, which may be attributable to the stress caused by the regular collection of blood.

### RT-nPCR detection of HEV RNA in rabbit sera and feces

All the sera and feces from the rabbits in the control group were negative for HEV RNA. Viremia was detected in only one rabbit (72B) at 72 dpi (Table 1). The feces of two rabbits (14B, 14D) in the inoculated group were positive for HEV RNA from 4 or 7 dpi to 14 dpi. From 21 to 72 dpi, the feces of three rabbits (72A, 72C, 72D) were temporarily positive, whereas the feces of only one rabbit (72B) was constantly positive for HEV RNA to the end of the study (Table 1).

**Table 1:** Samples of rabbits detected for HEV RNA by RT-nPCR.

Samples	Samples of rabbits detected for HEV RNA by RT-nPCR at days post-inoculation (dpi)											
	0	1	4	7	14	21	28	35	42	52	62	72
**serum**	**-**	**-**	**-**	**-**	**-**	**-**	**-**	**-**	**-**	**-**	**-**	**72B**
**feces**	**-**	**-**	**14B**	**14B,14D**	**14B,14D**	**72B**	**72B,72A,72C**	**72B,72D**	**72B**	**72B**	**72B**	**72B**
**Liver**	N	N	N	N	**14B,14D**	N	N	N	N	N	N	**72B**
**Spleen**	N	N	N	N	**14B, 14D**	N	N	N	N	N	N	**72B**
**Lung**	N	N	N	N	**14D**	N	N	N	N	N	N	**72B**
**Kidney**	N	N	N	N	**-**	N	N	N	N	N	N	**72B**
**Lymph node**	N	N	N	N	**14B, 14D**	N	N	N	N	N	N	**72B**
**Duodenum**	N	N	N	N	**14D**	N	N	N	N	N	N	**72B**
**Jejunum**	N	N	N	N	**-**	N	N	N	N	N	N	**72B**
**Ileum**	N	N	N	N	**14B,14D**	N	N	N	N	N	N	**72B**
**Sacculus rotundus**	N	N	N	N	**-**	N	N	N	N	N	N	**72B**
**Appendix**	N	N	N	N	**-**	N	N	N	N	N	N	**72B**

N: samples not detected by RT-nPCR; -: HEV RNA negative by RT-nPCR; 14B, 14D, 72A, 72B, 72C and 72D: HEV RNA positive rabbit number by RT-nPCR.

### ELISA determination

**Figure 1:**
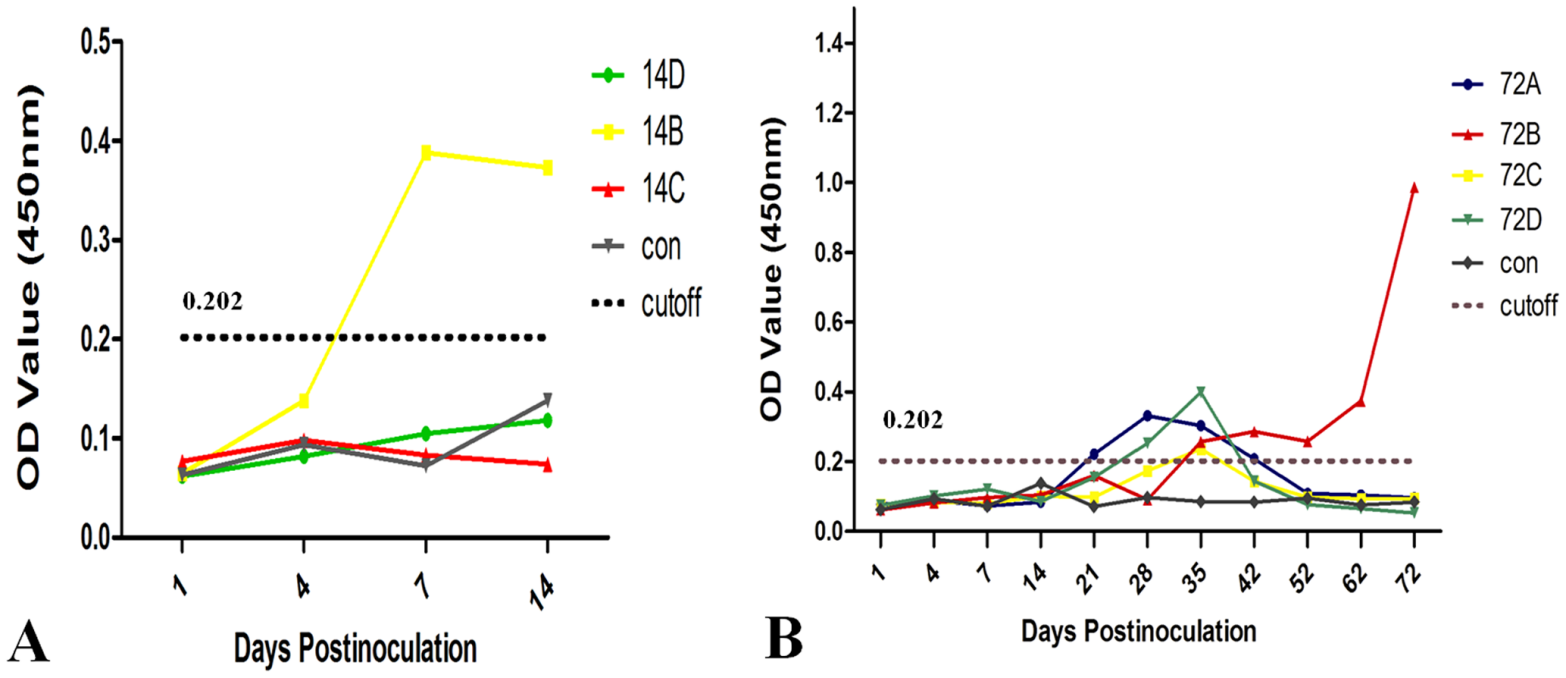
Serum HEV antigen tested by ELISA on various days postinoculation (dpi). **A** Serum HEV antigen tested by ELISA at 14“control”. Only one rabbit (14B) became positive for the HEV antigen. **B** Serum HEV antigen tested with an ELISA at 72 dpi. The HEV antigen showed temporary or constant elevation. The rabbits in the inoculated group were designated 72A, 72B, 72C, and 72D, and all the rabbits in the control group were designated “control”.

The rabbits in both the control group and the inoculated group remained negative for anti-HEV IgM and IgG antibodies throughout the study. However, one rabbit (14B) was positive for serum HEV antigen from before 7 dpi to 14 dpi (Fig. 1A). Four rabbits in the inoculated group were antigen positive from 21 dpi, but three of the four rabbits (72A, 72C, 72D) then became antigen negative for 2 or 3 weeks after inoculation. Only one rabbit (72B) remained antigen positive, and with an increasing titer, to the end of the study (Fig. 1B).

### RT-nPCR for HEV RNA in rabbit tissues

All the tissues from the rabbits in the control group tested negative for HEV RNA. At 14 dpi, the liver, spleen, ileum, and lymph-node samples from only two rabbits (14B, 14D) with HEV-RNA-positive feces were positive for HEV RNA. HEV was detected in the lung and duodenum of one of these two rabbits (14D). At 72 dpi, HEV was detected in all the tissues collected from the rabbit (72B) whose feces was constantly positive for HEV RNA, whereas the results were negative for the transiently HEV-positive rabbits (Table 1).

### Immunohistochemical staining for HEV antigen in rabbit tissues

**Figure 2:**
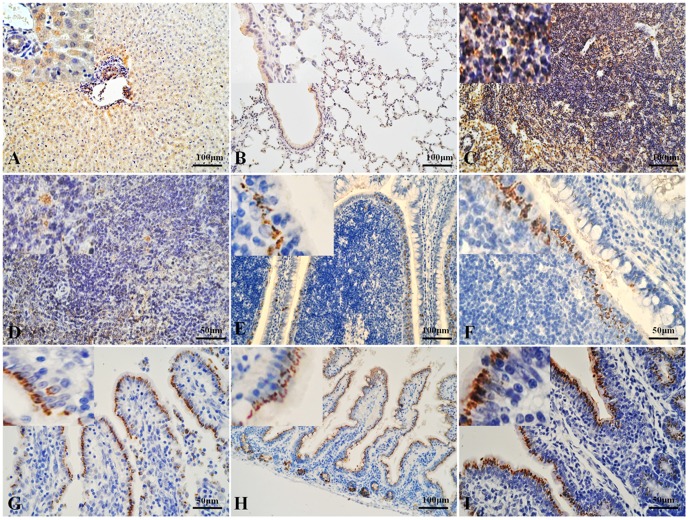
HEV antigen localization in inoculated rabbit tissues detected with immunohistochemical staining. A: Liver. The positive (yellow) signal for HEV antigen is distributed in the hepatocytes and epithelium of the bile ducts. B: Lung. The positive (yellow) signal for HEV antigen is distributed in the epithelium of the bronchioles and alveoli. C: Lymph node. The positive (yellow) signal for HEV antigen is distributed in the cortex and medulla. D: Spleen. The positive (yellow) signal for HEV antigen is distributed in various cells of the spleen. E, F: SR and appendix, respectively. The positive (yellow) signal for HEV antigen is distributed in the FAE of the SR and appendix. G, H, I: Duodenum, jejunum, and ileum, respectively. The positive (yellow) signal for HEV antigen is distributed in the mucosal epithelium.

**Figure 3:**
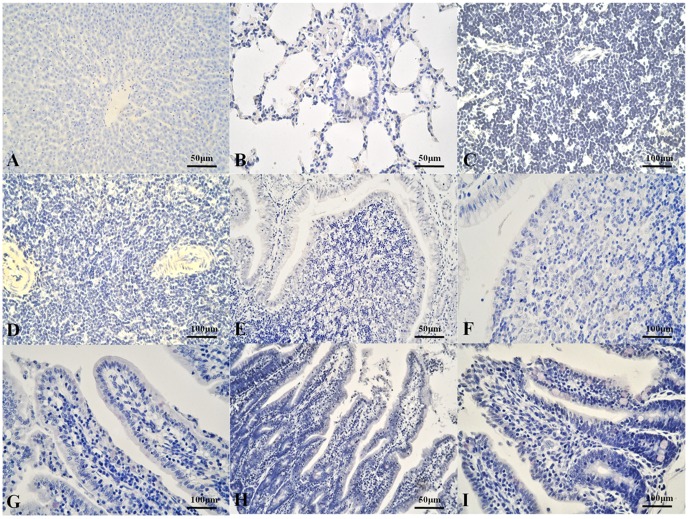
HEV antigen localization in rabbit tissues of control group detected with immunohistochemical staining. No positive (yellow) signal in rabbit tissues of control group. A: Liver. B: Lung. C: Lymph node. D: Spleen. E, F: SR and appendix, respectively. G, H, I: Duodenum, jejunum, and ileum, respectively.

With immunohistochemical staining, HEV antigen was detected in the duodenums, jejunums, ileums, SR, and appendices of two rabbits (14B, 14D) in the inoculated group at 14 dpi and in one rabbit (72B) at 72 dpi, whereas antigen was detected in the livers, spleens, lungs, and lymph nodes of all the rabbits in the inoculated group at 14 dpi and 72 dpi. Positive signals were observed in the cytoplasm of hepatocytes and biliary epithelial cells, the epithelium of the bronchioles and alveoli, various cells in spleen and lymph node, the intestinal epithelium, and the follicle-associated epithelium (FAE) of the SR and appendix (Fig. 2) [24]. We detected no positive signals in any tissue in the negative group or in the kidneys of the inoculated group (Fig. 3).

## Discussion

HE is recognized as a zoonosis because HEV has been detected in different kinds of animals, including the pig, boar, deer, chicken, rabbit, and rat [7]–[9], [11], [25]. Rabbit HEV was first discovered in China [11]. Ma et al. infected rabbits intravenously with first-generation and second-passage rabbit HEV, and showed that the second-passage rabbit HEV challenge, with a higher dose of virus, caused more severe hepatitis [16]. Therefore, we used as the inoculum the second passage of a rabbit HEV strain that had been collected from a positive fecal sample.

In contrast to the intravenous injections reported previously, we infected the rabbits intraperitoneally. Although HEV is considered to be transmitted via a fecal–oral route [3], it is difficult to infect with humans with HEV orally in experimental conditions [26]. Intraperitoneal infection may be a slower process than intravenous infection which delivers HEV directly into the blood. After intraperitoneal injection, the virus might be absorbed by the veins of the mesentery and then collected in the portal vein of the liver, which is similar to the absorption pathway after gavage.

HEV RNA was first detected in the rabbit feces at 4 dpi and persisted until 72 dpi, and thus appeared earlier than in a previous study of rabbits injected intravenously (detected 7 days post-injection) [16]. During the period from 21 to 42 dpi, three rabbits were temporarily positive, which is similar to the self-limiting symptoms observed in humans infected with HEV [27]. Interestingly, the same rabbits became temporarily antigen positive during this period, which might indicate that in short-term infections, fecal HEV RNA and HEV antigen appear synchronously. In contrast, in the constantly infected rabbits, HEV antigen was detected later than the fecal HEV RNA.

All rabbits were negative for anti-HEV IgM and IgG antibodies throughout the experimental period. In previous studies of other animals, serum antibodies have often been detected before 4 or 5 weeks [17], [18], [28], [29]. In rabbits experimentally infected with HEV, the antigen appeared about 2–8 weeks earlier than antibodies in most groups [16]. This might indicate that the antibody requires a longer period of stimulation before the animal becomes antibody positive. In our study, only one rabbit was antigen positive before 14 dpi, showing antigen at 4–7 dpi, and was killed at 14 dpi. In other rabbits, the antigen was expressed at low levels from 14 dpi to 72 dpi, and lasted for only a short time. Therefore, there may have been insufficient time to produce HEV-specific antibodies.

Previous studies have shown that HEV RNA might be replicated in the liver and many extrahepatic tissues in different animals [17], [30], [31]. In our study, we detected both viral RNA and viral antigen in different organs, especially in the three regions of the small intestine and the characteristic gut-associated lymphoid tissue (GALT)—the SR and appendix in the rabbit [24]. In our study, HEV RNA was detected in the liver, spleen, lung, lymph node, duodenum, and ileum of two inoculated rabbits at 14 dpi. Viral RNA was also detected in all the tissues collected from one constantly infected rabbit at 72 dpi. We conclude that HEV RNA can exist and might be replicated in extrahepatic organs, during acute and constant infection. HEV antigen was observed immunohistochemically. Antigen signals were only observed in the intestine and GALT of the fecal-HEV-positive rabbits at 14 dpi and 72 dpi. Antigen-positive signals were detected in the other organs (except the kidney) of all the inoculated rabbits. These results indicate that HEV antigens may be present in extrahepatic tissues, other than the intestine, during any part of the infection period, whereas the intestinal antigen might be related to the fecal virus. The antigen was also detected in more tissues and more rabbits than was HEV RNA. This implies that HEV antigen is a more sensitive index of infection than HEV RNA, or that the titer of HEV RNA was below the level of detection [12]. It is noteworthy that the intestine and GALT may play important roles in HEV infection.

## Conclusions

Rabbits were successfully infected with rabbit HEV via an intraperitoneal route. In HEV infected rabbits, HEV RNA and antigens distributed in different organs according to the different infection types. These data provide valuable information for future research into the pathogenesis of HEV.
